# Three-Dimensional Printed Biomimetic Robotic Fish for Dynamic Monitoring of Water Quality in Aquaculture

**DOI:** 10.3390/mi14081578

**Published:** 2023-08-10

**Authors:** Xiaojun Chen, Dejin Li, Deyun Mo, Zaifu Cui, Xin Li, Haishan Lian, Manfeng Gong

**Affiliations:** School of Mechanical and Electronic Engineering, Lingnan Normal University, Zhanjiang 524048, China

**Keywords:** bionic robotic fish, water quality detection, 3D printing

## Abstract

The extensive water pollution caused by production activities is a key issue that needs to be addressed in the aquaculture industry. The dynamic monitoring of water quality is essential for understanding water quality and the growth of fish fry. Here, a low-cost, low-noise, real-time monitoring and automatic feedback biomimetic robotic fish was proposed for the dynamic monitoring of multiple water quality parameters in aquaculture. The biomimetic robotic fish achieved a faster swimming speed and more stable posture control at a swing angular velocity of 16 rad/s by using simulation analysis. A fast swimming speed (0.4 m/s) was achieved through the control of double-jointed pectoral and caudal fins, exhibiting various types of movements, such as straight swimming, obstacle avoidance, turning, diving, and surfacing. As a demonstration of application, bionic robotic fish were placed in a lake for on-site water sampling and parameter detection. The relative average deviations in water quality parameters, such as water temperature, acidity and alkalinity, and turbidity, were 1.25%, 0.07%, and 0.94%, respectively, meeting the accuracy requirements for water quality parameter detection. In the future, bionic robotic fish are beneficial for monitoring water quality, fish populations, and behaviors, improving the efficiency and productivity of aquaculture, and also providing interesting tools and technologies for science education and ocean exploration.

## 1. Introduction

Water quality monitoring is of great importance in protecting water resources and promoting aquaculture. With the rapid development of intensive aquaculture, the large-scale water pollution caused by production activities has become a key concern for the aquaculture industry [1,2]. The water quality used in aquaculture directly affects the quality of aquaculture products. However, aquaculture farmers often do not pay enough attention to water quality monitoring and rely solely on their own experience [3]. Aquaculture production is often greatly affected by human factors and can easily lead to the phenomenon of pond eutrophication [4]. In order to obtain accurate information on the dynamic changes of pollutants in water in a timely manner, analyze pollution conditions in a timely manner, and study the diffusion, transfer, and transformation laws of pollutants, it is necessary to adopt and develop intelligent automatic detection technology [5,6,7,8,9].

The conventional methods of water quality monitoring are mainly carried out by manual operation, usually involving timed and fixed-point sampling at certain sections or monitoring points in water bodies, followed by laboratory analysis or on-site measurement [10,11]. Another fixed-point floating device detection method had fixed laying points and a high cost, and could not detect the water quality status of different areas and different water layers. Due to the limited number of sampling points and low frequency of monitoring, the accuracy and timeliness of the data obtained are poor, and the real-time monitoring of water quality in the water body cannot be achieved [12]. Additionally, the high cost and labor-intensive process make it challenging to achieve the real-time monitoring of water bodies. Therefore, developing a low-cost, real-time monitoring platform that can automatically provide feedback on results is crucial for the high-quality development of aquaculture.

Underwater vehicles can be divided into two categories: remotely operated underwater vehicles (ROUVs) and autonomous underwater vehicles (AUVs) [13]. ROUVs are large in size and operated remotely by operators, primarily used for life-saving purposes [14]. AUVs, propelled by propellers, achieve unmanned and fully automated operations, widely used in defense and military operations, oceanic exploration, and ecological restoration [15]. As conventional underwater monitoring devices, both ROUVs and AUVs suffer from drawbacks such as a high cost, specialized requirements, and high entry barriers [16]. In recent years, bionic robotic fish have emerged as a promising solution for water quality monitoring, attracting significant attention from researchers. These robotic fish have the ability to imitate the swimming behavior of real fish, making them highly maneuverable and stable in aquatic environments [17,18]. Compared to monitoring equipment propelled by propellers, bionic robotic fish are more flexible, efficient, low-polluting, and non-intrusive, allowing for water quality monitoring in different aquatic environments [19,20]. By utilizing machine fish as a carrier and combining bionics with water quality detection technology, the detection technology can be made more autonomous and intelligent, effectively solving the problem of the low efficiency of traditional detection technology. A high polymer electrolyte ion exchange membrane was coated onto a metal plate of a bionic fish fin, and an external electric field was applied to produce a swimming motion similar to that of an electric eel [21]. Karimanzira et al. [22] equipped underwater robots with conductivity and dissolved oxygen sensors to achieve large-scale water quality monitoring and underwater cruising. However, due to the large size of the equipment, it cannot work in complex terrain and environments with too many underwater organisms. Wang et al. [23] designed a simulation machine fish water quality detection system based on ZigBee and GPRS. Although they introduced a machine fish cruising system, the cruising path lacked mathematical models support, and the movement speed was slow. Zhang et al. [24] developed a framework-type cable-free underwater robot equipped with a video monitoring system, but the design lacked flexibility and could disturb underwater populations. In general, there is as lack of water quality testing equipment that does not disturb underwater and is suitable for various complex environmental work. Although research on bionic robotic fish is still ongoing, there is a lack of literature reports on their practical applications, especially in the dynamic detection of multiple parameters in aquatic environments [25]. Despite these challenges, ongoing research in the field of bionic robotic fish shows promise for the development of practical and efficient water quality monitoring systems. By overcoming current limitations and addressing the need for flexibility, non-disturbance, and multi-parameter detection capabilities, bionic robotic fish can play a vital role in improving water quality monitoring in various complex environmental settings.

With the development of intelligent sensing technology, the integration and functionality of bionic robotic fish have been greatly improved, providing a new approach to solving the problem of measuring multiple parameters of water quality. Here, a bionic robotic fish based on dual servo control was proposed, which was used to detect multiple parameters such as water temperature, pH value, and turbidity in aquatic environments. The fish body of the bionic robotic fish was manufactured in one step using 3D printing technology and integrates multiple water quality monitoring sensors and motion controllers. The bionic robotic fish mimics the swimming behavior of natural fish by using a control method that combines the pectoral fins and caudal fin, allowing it to perform automatic cruising and obstacle avoidance in the target water environment. The collected water quality parameters are transmitted to the host computer through a wireless sensor network, enabling the real-time monitoring and management of the water quality parameters. Compared to traditional water quality monitoring methods, it has the characteristics of convenience and timeliness, which meets the market demand for water quality monitoring. The use of intelligent biomimetic robotic fish will bring new water quality detection solutions to the aquaculture industry. In the future, our biomimetic robotic fish will play an important role in aquaculture, water pollution protection, and marine exploration.

## 2. Materials and Methods

### 2.1. Experimental Materials

PLA consumables were purchased from Rambo brand, which belongs to Cixi Rambo Printing Consumables Co., Ltd. (Cixi, China). Waterproof glue RF705K was purchased from Shenzhen Shenchuang Electronics Co., Ltd. (Shenzhen, China). Silica gel film was purchased from Mai Ji Hardware Co., Ltd. (Shanghai, China) Two-axis steering engine was purchased from Shenzhen Sky Star Technology Co., Ltd. (Shenzhen, China). Water quality sensor was purchased from Chongqing Shenhe Intelligent Technology Co., Ltd. (Chongqing, China). The PH standard solution and PH test paper were purchased from Lichen Technology Co., Ltd. (Taipei, Taiwan). The turbidity standard solution of turbidity detection reagent was purchased from Hangzhou Qiwei Instrument Co., Ltd. (Hangzhou, China). The temperature detector was purchased from Shandong Senteng Mechanical Equipment Co., Ltd. (Jining, China)

### 2.2. Manufacturing and Assembly of Bionic Robotic Fish

The structure of the bionic robot fish was modeled using SolidWorks software (2018 version, SolidWorks Corp., Waltham, MA, USA), including the fish body shell, pectoral fin module, water quality sensor installation module, tail fin and fish tail swing module, and waterproof module. FDM (fused deposition modeling) is a 3D printing technology suitable for prototyping, small batch production, and customization of products. It utilizes thermoplastic materials to create objects by extruding and layering the material from a nozzle. The 3D printing technology was used to fabricate the body parts of the biomimetic robotic fish in a single integrated form. The bionic robot model was converted into .STL format graphic file and imported into a slicing software (V3.1) for slicing. After printing, the parts were post-processed by using polishing fluid and polishing pen on the surface. Then, the electronic components and sensors were arranged in their designated positions to complete the assembly of the entire bionic fish. The assembly exploded view of the robotic fish is shown in Figure S1 (Supplementary Materials). A triple waterproof design was adopted to address the waterproofing issues of the mechanical structure and electronic components in the bionic fish. Firstly, waterproof silicone adhesive was used to seal the electronic components inside the fish body to prevent water leakage and electronic component short circuits. The fish body contour was sealed and filled with hot melt glue between the parts, and waterproof tape was used to bond and fix it, preventing water infiltration and sand erosion. Finally, a biomimetic fish skin was wrapped around the caudal fin to prevent water from entering the side of the fin during its movement.

### 2.3. Propulsion and Hydrodynamic Simulation of Bionic Fish

When analyzing the propulsion of a bionic robotic fish, the force of the fish’s tail fin is divided into added mass force and viscous resistance. When the robotic fish swims in water, it will carry the surrounding fluid to move together, which can be regarded as an increase in the mass of the fish’s body. The increased mass is the added mass. The bionic robotic fish’s tail fin moves according to a cosine function. The fish head is subject to the force *F* and moment *M* from the fish tail, satisfying the Newton–Euler equation:

$$F=-m_{t}\dot{V}\;M=-J_{t}{\dot{\Omega }}_{t}-\Omega_{t}*J_{t}\Omega_{t}+r_{bt}*F$$

In this equation, *m_t_* and *J_t_* are the mass and moment of inertia of the fish head, respectively. The velocity of the fish head is *V_t_*, the angular velocity is Ω*_b_*, and *r_bt_* represents the distance from point *b* to point *t*.

SolidWorks was used to create a two-dimensional geometric water domain and the robotic fish model. The fluid configuration was assumed to be a boundary flow, with the fish body boundaries and water domain boundaries. To accelerate the convergence speed of the calculations, a pressure point constraint was applied at one point in the water domain. The fish head was set as a fixed constraint, while a sine wave pressure constraint was applied to the fish tail to simulate its oscillation. A sine wave boundary load was also set and, in the definition, a “piecewise function” pw1(t) was defined with t as the variable and a function value of 10. An “analytic function” an1(t) was added to the definition, with t as the variable in seconds. The function expression was set as “pw1(t)sin(wt)*10”, where w is the angular frequency defined globally in the “parameters” section.

In geometric grid partitioning, a structured grid can be used for the flow around a 2D fish tail. The total number of grids was 10,480, with 350 grid points distributed circumferentially along the fish body and 106 grid points on the tail fin. The grid growth rate was 1.08. After the grid partitioning, parameterized scans with different angular frequencies were set: range (2,2,8). Different values of the angular frequency were chosen to compare the motion state of the fish tail and the velocity and pressure changes of the laminar flow.

The water dynamics simulation was conducted using the COMSOL 6.1 finite element method (FEM) for the bionic fish. When simulating the two-dimensional tail fin swing, it was assumed that the water flow velocity was 0. The bionic fish relied on its tail fin to provide periodic swing for the entire fish, thus achieving actions such as forward movement and turning. Here, a simple vibration mode function was established:

$$\varphi (x)=(\frac{X}{X_{max}})2^{\;}\times \;y_{max}$$

Here, *X* is the coordinate of a point on the caudal fin; *X_max_* is the maximum value of x; and *y_max_* is the maximum displacement of *y*. The unit is m.

In the calculation domain, the SST k-w turbulence model was used for steady-state flow simulations. The simulation time was 5 s with a time step of 0.02 s. The boundary conditions were set consistent with the grid boundary conditions, and the initial condition was set with a water flow velocity of 0.

### 2.4. Bionic Robotic Fish Swimming Test

First, the assembled bionic robotic fish underwent an indoor swimming performance test. The fish was fully immersed in a water environment for 5 min to test its waterproof performance. If any leaks occur, the fish will need to be disassembled and undergo waterproof treatment. Next, the fish underwent indoor swimming performance tests in a swimming pool, including straight swimming, turning, and diving. Finally, we took the robotic fish to an outdoor lake to conduct water quality parameter testing experiments. During the water quality monitoring process, we pre-configured the sampling path and sampling time in the program. When the robotic fish reaches the designated location, the sensors started conducting water quality sample tests. The monitoring data were then transmitted to a mobile app for real-time display of the water quality condition.

## 3. Results and Discussion

### 3.1. Three-Dimensionally Printed Bionic Machine Fish

**Figure 1:**
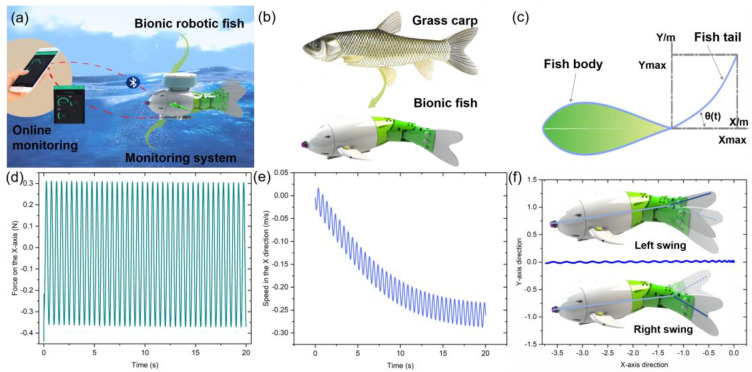
Bionic fish. (**a**) schematic diagram of water quality detection; (**b**) schematic diagram of bionic fish and natural fish; (**c**) two-dimensional motion model; (**d**) bionic fish motion model; (**e**) distribution of force with displacement, where the X-axis represents the thrust, and −X represents the forward direction; (**f**) swimming trajectory in the X-axis direction.

Here, a bionic robotic fish integrated with multiple parameter sensors was proposed, which can be used for water quality monitoring in complex environments such as rivers, lakes, and fishponds (Figure 1a). The robotic fish imitated the external contour of grass carp in nature and was assembled with 3D-printed components, including a fish body shell, pectoral fin module, water quality sensor module, tail fin, and fish tail swing module (Figure 1b). The overall size of the robotic fish was 48 (L) × 219 (W) × 232 mm (H) (Figure S2, Supplementary Materials). The water quality sensor module integrates multiple parameter target detection, including a temperature sensor, pH sensor, and turbidity sensor.

To verify that the bionic robotic fish could swim smoothly, a motion model was established to analyze the propulsion force during the fish’s swimming process (Figure 1c). It was assumed that the fish head of the bionic robotic fish was not fixed and propulsion force was generated by the swinging of the tail fin. As shown in Figure 1d, the force received by the fish was offset toward the X-axis direction, indicating the presence of propulsion force. The distribution of force along the X-axis was relatively uniform, indicating a stable propulsion force. Figure 1e shows that, when the force was generated, the speed was concentrated along the X-axis direction and the speed direction was consistent with the force direction, making the fish head controllable in the forward direction. Simulation results show that the force remained close to the axis of the fish body, thus producing propulsion force and causing the fish to swim in a straight line, indicating that the bionic robotic fish could swim normally. As shown in Figure 1f, the robotic fish could swim steadily along a straight line without any significant deviation in direction.

### 3.2. Water Hydrodynamic Analysis of the Bionic Robotic Fish

The bionic robotic fish was designed based on the characteristics of fish biology, with a body shape and pectoral fin similar to the grass carp. When swimming, the bionic robotic fish has different swimming speeds and swimming postures with different frequencies and amplitudes of its tail fin [26]. By adjusting the frequency and amplitude of tail fin swing, the swimming speed and posture of the bionic robotic fish can be controlled to enable it to move freely in different aquatic environments. In order to study the hydrodynamic characteristics of the bionic robotic fish, an FEM flow field module was used for the simulation.

The bionic robot fish relies on the periodic swinging of its tail fin to provide propulsion and achieve movements such as forward motion and turning. According to the relationship between force and displacement, when the force was negative, it provided the propulsive force that drives the robot fish forward, and when the force was positive, it was considered as the resistance that impedes the forward motion of the robot fish. In this model, the effect of the force generated in the X-axis direction during tail fin swinging on the robot fish’s movement was considered. The tail fin swinging angle of natural fish has a certain range. By setting the tail fin swinging angular velocity to 4 rad/s, 12 rad/s, and 16 rad/s, respectively, the movement of the tail fin was analyzed.

**Figure 2:**
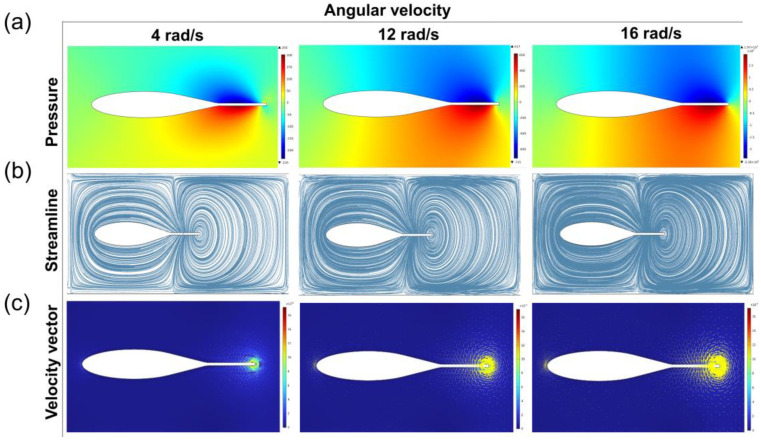
Simulation of the swimming of bionic fish: (**a**) pressure distribution, (**b**) streamline distribution, and (**c**) velocity vector map.

Figure 2a shows the pressure distribution of the tail fin swinging at different angular velocities. When the tail fin swings to the right direction of forward motion, a wide positive pressure area was generated on the right side of the tail fin, and a wide negative pressure area was generated on the left side. The machine fish relies on the pressure difference between these areas to generate forward thrust [27]. As the angular velocity of the tail fin swing increases, the thrust force exerted on the machine fish during swimming will increase, particularly with a significant increase from 12 rad/s to 16 rad/s. When the tail fin swing angular velocity is 16 rad/s, the whole body is almost in the pressure difference zone. Therefore, a better performance can be achieved when the tail fin swinging speed is 16 rad/s.

In Figure 2b, when the tail fin swing speed of the robotic fish is 4 rad/s, the streamlines are distributed around the fish. When the tail fin swing speed is 12 rad/s, the streamlines mainly distribute above the fish body. When the tail fin swing speed is 16 rad/s, the streamlines are denser than those at 12 rad/s. The geometric tangent of each point on the streamline is the velocity direction of the fluid element at that point [28]. Under the same flow field, the density of the streamline distribution caused by the three different swing speeds is different. The denser the streamline distribution, the higher the flow velocity and the greater the thrust under the interaction force. In Figure 2c, there is a similarity between the velocity vector distribution and the streamline distribution under different swing speeds. With an increase in the swing speed, the streamline distribution and velocity vector distribution are dense in the region close to the tail fin.

**Figure 3:**
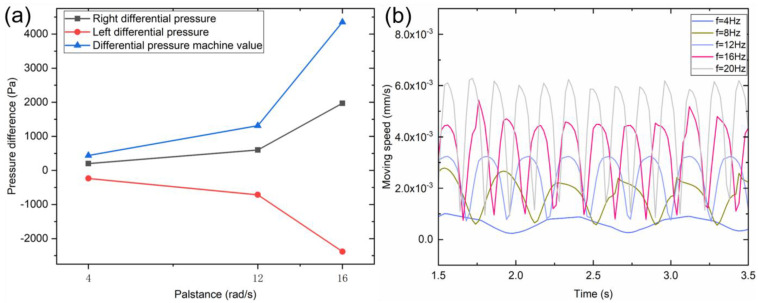
(**a**) relationship between the extreme value of pressure difference and the frequency of swimming; (**b**) variation in swimming velocity over time at different swimming frequencies.

From a pressure perspective, a swinging speed of 16 rad/s is more appropriate for a bio-inspired robotic fish when fast movement is required. However, this also leads to a larger range and magnitude of pressure differences around the fish body, causing instability during swimming (Figure 3a). If the goal is to use the bio-inspired robotic fish for tasks such as reconnaissance, choosing a lower oscillation speed can make the fish body more stable and easier to control. Figure 3b shows the relationship between the water flow velocity at the tail fin and time at different oscillation frequencies. Based on the flow lines and velocity vector distribution, the flow lines are relatively concentrated at the tail fin, resulting in a lower velocity and smaller propulsion force at a swinging speed of 4 rad/s. When the swing speed is 16 rad/s, the streamlined distribution is wider and dense. The speed vector distribution brings a large speed difference and generates a large thrust. Therefore, the swimming speed of bionic machine fish is faster.

### 3.3. Bionic Fish Propulsion Experiment in Water

**Figure 4:**
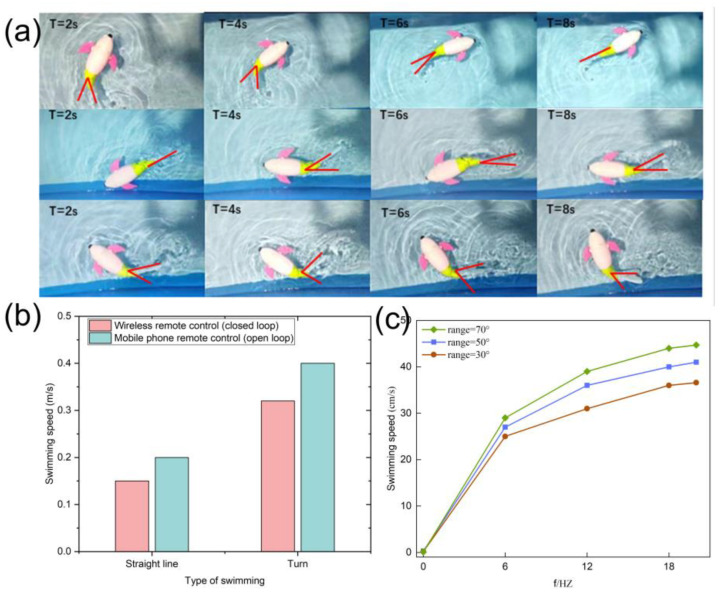
Bionic fish swimming behavior: (**a**) swimming behaviors such as straight swimming, turning, and obstacle avoidance; (**b**) swimming speed of the remotely controlled robotic fish via wireless remote control and mobile phone during straight swimming and turning; (**c**) relationship between swimming speed and swimming frequency at different swing angles.

During the process of water quality monitoring, the motion of the bionic robotic fish can be divided into three types: forward swimming, turning left and right, and diving and surfacing (Figure 4a). The robotic fish adopts a dual-joint propulsion mode for its tail fin, powered by a servo motor. The force used for the fish to swim forward mainly relies on the swinging module driving the tail fin to move left and right. By changing the swing amplitude of the tail fin, the fish can change its swimming direction, such as turning. In addition, a dual-axis servo motor was used to control the pectoral fin module to produce upward, downward, and parallel forces, thereby achieving the motion of diving and surfacing. These three types of motion enable the bionic robotic fish to swim freely in the water like natural fish (Video S1, Supplementary Materials).

As shown in Figure 4b, the swimming speed of the robotic fish during turning was faster than that during straight swimming under both wireless and infrared control. This was because the robotic fish needs to increase the swing angle of its tail fin to obtain a faster instantaneous speed when turning to avoid obstacles. The relationship between the swing angle range of the tail fin (30°, 50°, 70°) and the swimming speed was investigated, as shown in Figure 4c. As the tail fin swing angle increases, the swimming speed of the bionic robotic fish increases. Combined with the distribution of velocity vectors, when the swing angle velocity of the tail fin was the same, the larger the swing angle, the larger the generated vortex, resulting in a faster swimming speed of the robotic fish.

**Figure 5:**
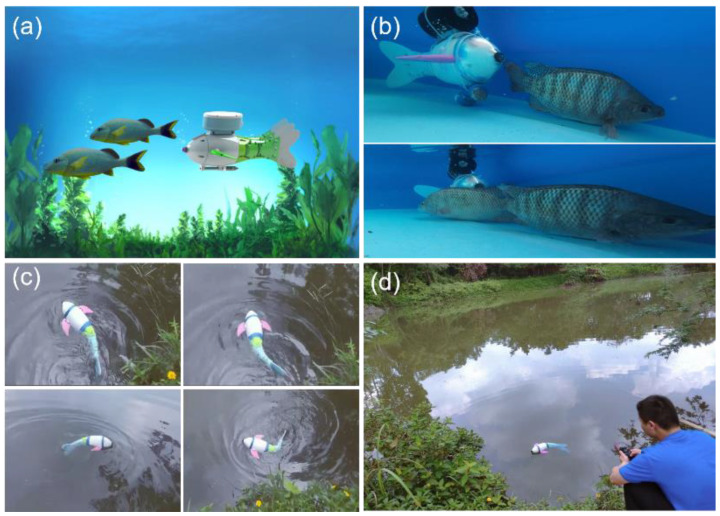
Field testing of bionic robotic fish swimming behavior: (**a**) schematic of fish shoaling behavior; (**b**) adaptability of the bionic robotic fish; (**c**) on-site swimming behavior testing; (**d**) wireless and infrared control.

The swimming behavior of robotic fish in water can affect the swimming behavior of natural fish [29]. We placed fish in an experimental fish tank to observe the swimming behavior of the bionic fish (Figure 5a). As shown in Figure 5b, the bionic fish swam behind the natural fish, and the natural fish did not feel scared because of the bionic fish. When they swam face-to-face, the natural fish did not exhibit abnormal behavior, demonstrating the high adaptability of bionic fish in fish schools (Video S2, Supplementary Materials). Additionally, the interaction between robotic fish and natural fish can make natural fish more active, which is beneficial for improving the quality of aquaculture products [30].

To demonstrate the swimming ability of the bionic robotic fish in the wild, we tested its swimming behavior in a lake. As shown in Figure 5c, the bionic robotic fish was able to swim forward, turn left and right, and dive and rise in the lake. There was no significant water wave disturbance, indicating that the fluid disturbance caused by swimming was small. The swimming behavior of the bionic robotic fish was normal under both wireless and infrared control (Figure 5d). Therefore, the aquatic environment does not have a significant impact on the autonomous swimming of the bionic robotic fish.

### 3.4. Application of On-Site Water Quality Monitoring

On-site water quality parameter testing is of utmost importance and significance to aquaculture. It enables the real-time monitoring of essential water quality parameters, ensuring a healthy and optimal environment for aquatic organisms. Rapid and accurate monitoring helps farmers to promptly identify deviations and take preventive or corrective measures to mitigate any potential problems. Real-time monitoring also helps in ensuring a stable and favorable environment throughout different stages of aquaculture production. In summary, turbidity, temperature, and pH are crucial water quality parameters with significant impacts on aquaculture. By regularly monitoring and managing these parameters, aquaculturists can optimize environmental conditions, promote healthy growth and reproduction, and ultimately ensure the overall success and sustainability of their aquaculture operations.

**Figure 6:**
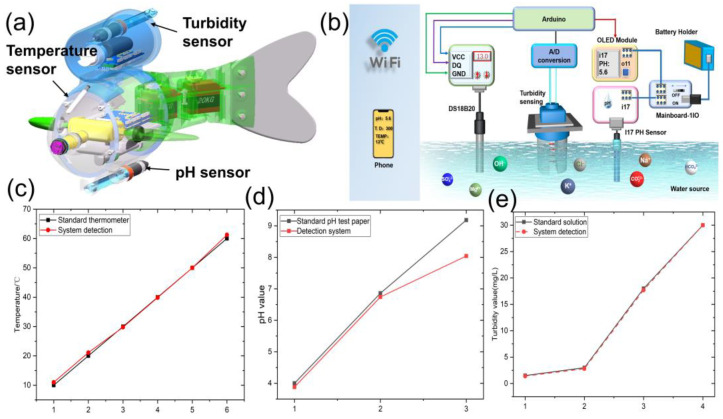
Schematic diagram of (**a**) the sensor arrangement structure and (**b**) the water quality detection system; (**c**–**e**) comparison of system testing and standard detection.

The bionic robotic fish was equipped with a variety of sensor parameters for detection, including temperature sensors, PH sensors, turbidity sensors, and infrared obstacle avoidance sensors (Figure 6a). The water quality detection data were transmitted to the Arduino microcontroller platform and then transferred to the upper computer in real-time through WiFi to achieve the purpose of real-time monitoring (Figure 6b). The water quality detection parameters through testing were standardized (Figure S3, Supplementary Materials). According to the principle of analog-to-digital conversion, parameters such as PH value, temperature, and turbidity show a linear relationship with voltage.

To verify the accuracy of the water quality detection system, the bionic fish water quality detection system was used to test the PH value, temperature, and turbidity, respectively. The data obtained from the water quality detection system were compared with the data obtained from standard PH test strips, standard thermometers, and standard turbidity solutions, and the results of the water quality detection system were found to be very close to the standard data (Figure 6c–e).

**Figure 7:**
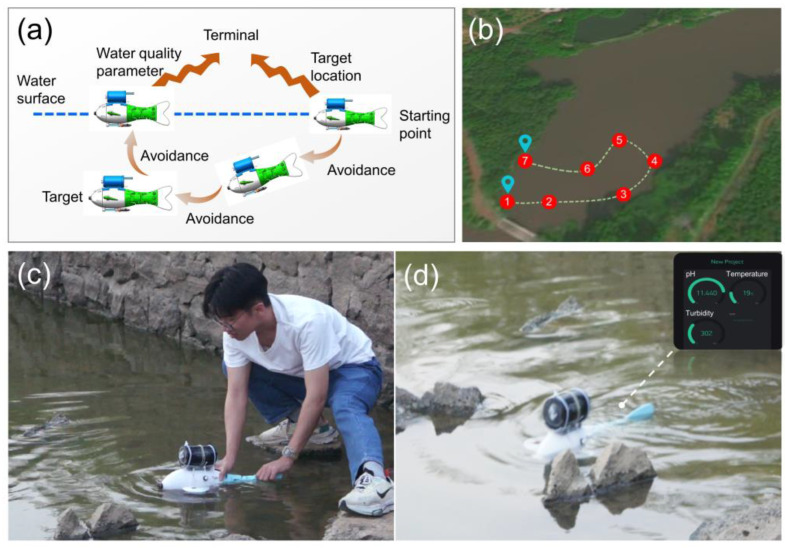
On-site monitoring of water quality parameters. (**a**) water quality parameter testing process, (**b**) sampling points for water quality, 1 is the starting point and 7 is the endpoint., (**c**) water quality sample collection site, (**d**) real-time water quality data monitoring.

The bionic robotic fish was placed in a lake for underwater navigation and water quality parameter collection experiments. Figure 7a shows the working process of the water quality detection bionic robotic fish. First, the robotic fish is placed on the water surface, and the target position is sent to the robotic fish’s wireless module from a mobile phone. After comparing the target position with its own position, the robotic fish swims toward the target point and collects water quality parameters through sensors when it arrives at the target location. The data are transmitted to the mobile phone in real-time through the wireless module. The robotic fish was used to test the water quality at seven testing points in the lake by swimming around (Figure 7b). Figure 7c shows the bionic robotic fish performing on-site water quality sampling. Through the wireless module, the water quality monitoring parameters are transmitted to the mobile phone interface and display real-time monitoring data (Figure 7d).

Water quality parameters usually include indicators such as water temperature, pH, dissolved oxygen, and turbidity [31]. These parameters can reflect the physical, chemical, and biological characteristics of water bodies, and are important indicators for evaluating water quality [32]. Through water quality parameter investigation, various substances’ concentration and distribution in water bodies can be monitored in a timely manner, the pollution situation can be understood, and measures can be taken in a timely manner.

According to the experimental results, there was a certain error between the measured values and the actual values of the bionic fish system (Table 1). The relative average deviation of water temperature, pH, and turbidity is 1.25%, 0.07%, and 0.94%, respectively. Compared with the previous literature [33], it has obvious advantages in measurement accuracy. According to a multi-factor analysis, the error was caused by the accuracy error of the measuring sensor itself and the differences in the sampling point environment, resulting in small-scale experimental errors between sampling points. By conducting multiple experiments and using algorithms to correct errors, it was possible to achieve the requirement of the online detection of multiple water quality parameters.

## 4. Conclusions

In this paper, a bionic robotic fish was proposed by using 3D printing technology, which integrates bi-directional control of pectoral and caudal fins. The fish was equipped with multiple water quality monitoring sensors, enabling the real-time sampling and monitoring of water samples. Field water quality parameter detection was conducted in lakes, and the results show that the system meets the requirements of water quality parameter detection. Additionally, we will strive to improve the body structure and motion control algorithms of the fish to enhance its swimming performance and adaptability in diverse and challenging environments. In the future, we plan to expand the application scope of the robotic fish to areas such as aquatic biological research, intelligent aquaculture, and environmental monitoring, making significant contributions to sustainable water quality monitoring and ecological protection.

## Figures and Tables

**Table 1:** Water quality monitoring sampling data.

Samples	Water Temperature			Turbidity			Ph Value		
	Test Value	Actual Value	Error	Test Value	Actual Value	Error	Test Value	Actual Value	Error
1	20.21	20.73	0.52	100.59	100.79	0.2	8.447	8.55	0.103
2	20.332	20.712	0.38	101.56	101.775	0.215	8.45	8.569	0.119
3	20.422	20.644	0.242	105.95	106.173	0.223	8.489	8.587	0.098
4	20.51	20.407	0.103	120.89	121.09	0.2	8.502	8.603	0.101
5	20.553	20.468	0.085	121.32	121.526	0.206	8.507	8.623	0.116
6	20.597	20.8	0.203	130.85	131.109	0.259	8.548	8.674	0.126
7	20.6	20.903	0.303	133.89	134.151	0.261	8.561	8.693	0.132
8	20.662	20.821	0.159	140.96	141.235	0.275	8.575	8.705	0.130

## Data Availability

Not applicable.

## Supplementary Materials

The following supporting information can be downloaded at: https://www.mdpi.com/article/10.3390/mi14081578/s1, Figure S1: Main dimensions of robotic fish, (a) front view, (b) top view. The whole size is mm; Figure S2: Bionic machine fish structure explosion diagram; Figure S3: Water Quality Parameter Calibration Curve. Video S1: Swimming Behavior Experiment. Video S2: Lake Swimming Experiment.
