# Supplementary material for: Three-Dimensional Printed Biomimetic Robotic Fish for Dynamic Monitoring of Water Quality in Aquaculture
Source: Micromachines (Basel). 2023 Aug 10;14(8):1578. doi: 10.3390/mi14081578 (PMC10456635; doi:10.3390/mi14081578)
Supplement: Supplementary file 1 [file micromachines-14-01578-s001.zip › micromachines-2516428 - Supplementary file - Highlighted.pdf]

Supplementary Materials

3D printed biomimetic robotic fish for dynamic monitoring of water quality in aquaculture

Xiaojun Chen *, Dejin Li, Deyun Mo, Zaifu Cui, Xin Li, Haishan Lian and Manfeng Gong

School of Mechanical and Electronic Engineering, Lingnan Normal University, Zhanjiang, China

* Correspondence: chxj@lingnan.edu.cn

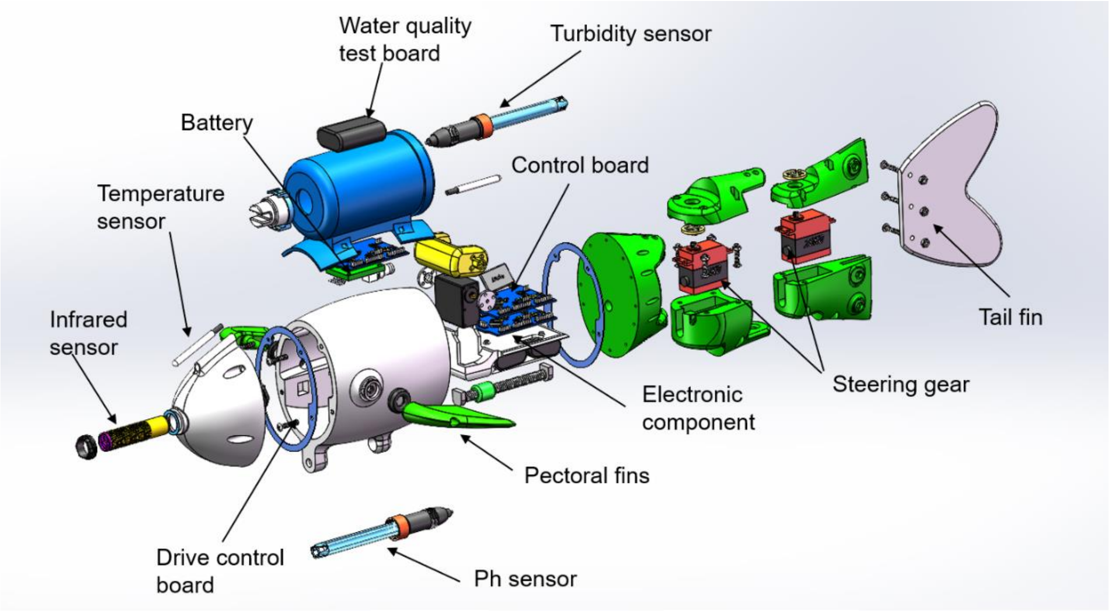

Figure S1. Bionic machine fish structure explosion diagram

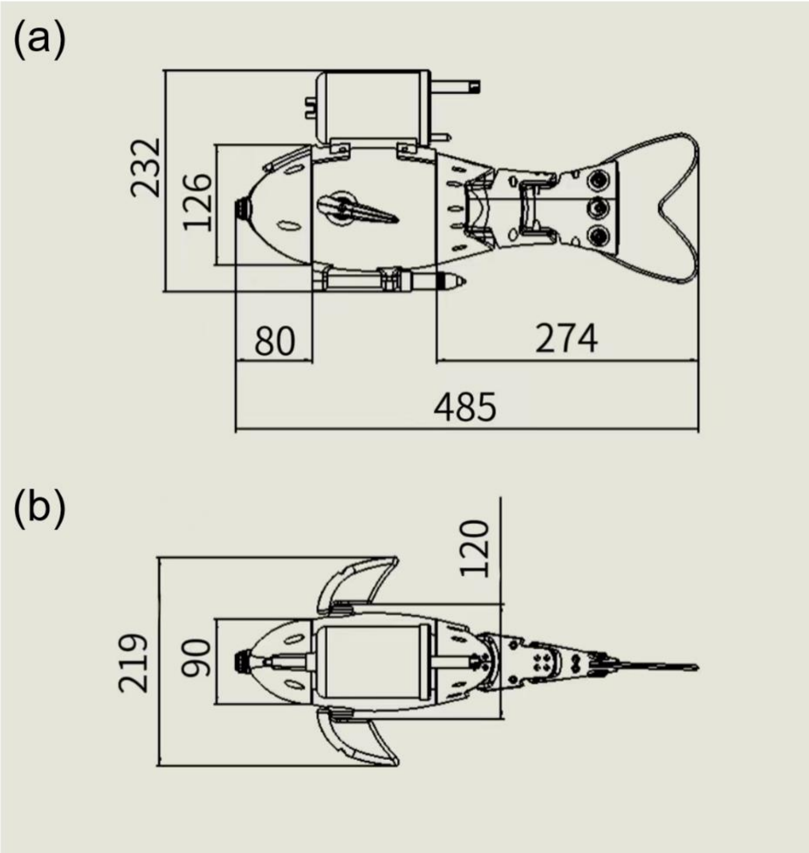

Figure S2. Main dimensions of robotic fish, (a) front view， (b) top view. The whole size is mm.

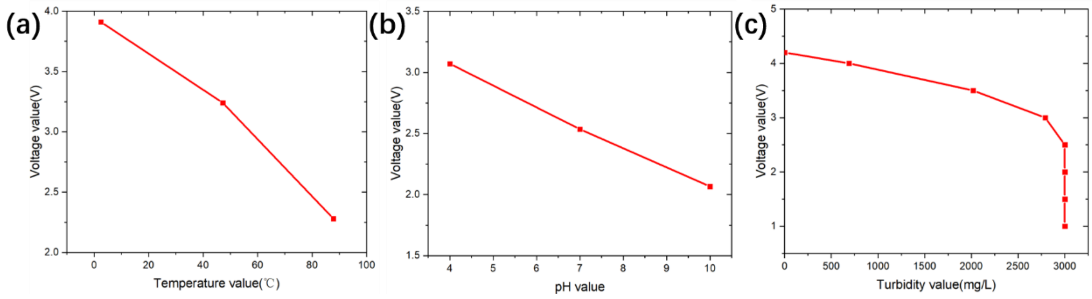

Figure S3. Water Quality Parameter Calibration Curve
